# Curing, Properties and EMI Absorption Shielding of Rubber Composites Based on Ferrites and Carbon Fibres

**DOI:** 10.3390/polym15040857

**Published:** 2023-02-09

**Authors:** Ján Kruželák, Andrea Kvasničáková, Michaela Džuganová, Lenka Hašková, Rastislav Dosoudil, Ivan Hudec

**Affiliations:** 1 Department of Plastics, Rubber and Fibres, Faculty of Chemical and Food Technology, Slovak University of Technology in Bratislava, Radlinského 9, 812 37 Bratislava, Slovakia; 2 Department of Electromagnetic Theory, Faculty of Electrical Engineering and Information Technology, Slovak University of Technology in Bratislava, Iľkovičova 3, 812 19 Bratislava, Slovakia

**Keywords:** manganese–zinc ferrite, nickel–zinc ferrite, carbon fibres, electromagnetic radiation, absorption shielding

## Abstract

In this work, magnetic soft ferrites, namely manganese–zinc ferrite, nickel–zinc ferrite and combinations of both fillers, were incorporated into acrylonitrile-butadiene rubber to fabricate composite materials. The total content of ferrites was kept constant—300 phr. The second series of composites was fabricated with a similar composition. Moreover, carbon fibres were incorporated into rubber compounds in constant amount—25 phr. The work was focused on investigation of the fillers on absorption shieling performance of the composites, which was investigated within the frequency range 1–6 GHz. Then, the physical–mechanical properties of the composites were evaluated. The achieved results demonstrated that the absorption shielding efficiency of both composite types increased with increasing proportion of nickel–zinc ferrite, which suggests that nickel–zinc ferrite demonstrated better absorption shielding potential. Higher electrical conductivity and higher permittivity of composites filled with carbon fibres and ferrites resulted in their lower absorption shielding performance. Simultaneously, they absorbed electromagnetic radiation at lower frequencies. On the other hand, carbon fibres reinforced the rubber matrix, and subsequent improvement in physical–mechanical properties was recorded.

## 1. Introduction

Rapid technological development in today’s modern society is connected with increasing demand for modern radio, electronic and communication devices but also local network and radar systems. Emission of electromagnetic waves from those devices results in potential for one electromagnetic signal to interfere with another. This negative effect is termed as electromagnetic interference (EMI). In communication devices (mobile phones, Bluetooth devices, computers), commercial appliances (TV sets, microwave ovens) and the automotive industry (electronic equipment), EMI deteriorates durability and proper functioning of integrated electrical circuits [1,2,3,4]. Experimental studies have demonstrated that long-lasting exposure to EMI can also pose a significant threat to biological lifeforms. Life-threatening conditions such as cancer, heart problems, asthma, insomnia, irritability and even miscarriages have been reported [5,6,7]. Thus, significant demand has resulted regarding eliminating or mitigating harmful EMI. Electromagnetic radiation shielding can be defined as reflection and/or absorption of EMI by material, which acts as a barrier against penetration of radiation [1]. 

In past decades, various conductive materials (such as metals, carbon-based fillers or MXene, etc.) or magnetic materials (metal oxides, ferrites, alloys, etc.) have been successfully used for mitigation or elimination of EMI mostly via magnetic or dielectric loss [8,9,10,11,12,13,14,15,16]. Metals and metallic composites belong to the oldest materials used for EMI shielding, but their utilization is limited by their high density, rigidity, poor mechanical flexibility, susceptibility to corrosion, difficult processability and high production costs [17,18]. Recently, carbon-based materials, such as graphite, graphene, carbon fibres or carbon nanotubes, have been successfully tested as EMI shields, mainly due to their high conductivity, chemical and thermal stability and tuneable electrical, optical and physical–mechanical properties [18,19,20]. Moreover, when incorporated into polymer matrices, they demonstrate higher or lower reinforcing character and contribute not only to enhanced EMI shielding performance but also improve their dynamic and physical–mechanical properties. 

The largest drawback of conductive materials used as EMI shields is that they suffer from impedance mismatching, which results in reflection of electromagnetic radiation rather than absorption, or they show narrow effective absorption frequency bandwidth [16,20,21]. Reflection shielding is unwanted as reflected plane waves can interfere with radiation emitted from neighbouring electronic sources, thus causing a secondary EMI effect. Therefore, carbon-based materials are often combined with low electrically conductive materials to improve the impedance match [18,20,22,23,24,25,26,27]. 

Magnetic responding materials have been demonstrated to achieve good EMI absorption capacity within wide frequency ranges [16,28]. To such materials belong magnetic soft ferrites. Ferrites are ceramic compounds composed of iron oxide (Fe_2_O_3_) chemically combined with one or more additional metallic elements. Ferrites demonstrate high permeability, high electrical resistivity, low eddy current loss, wide frequency range, shape versatility, low cost, etc. They are very important materials in the electronics industry. Ferrites are applied in various applications, such as magnetic sensors, magnetic recordings, electromagnetic radiation shielding, memory devices, mobile communication, electronic components, gyromagnetic devices, transformers, pollution control sensors, pigments, catalysis, etc. [29,30,31]. 

Polymers, with the exception of intrinsically conductive polymers, are typical electrical insulators. Therefore, they are not able to shield EMI. However, incorporation of suitable fillers into polymer matrices leads to fabrication of composites that can reach significant status among materials, demonstrating EMI shielding performance. Polymer composites have been deeply investigated as materials for EMI shielding applications due to their flexibility, stability, light weight, extensive absorption, corrosion resistance, ease of processing, etc. Investigation of polymer composites used as EMI shields that were fabricated by incorporation of carbon-based fillers and ferrites and in various polymer matrices has been the interest of many experimental studies [32,33,34,35,36,37,38,39]. Results have shown that such composites can effectively shield harmful EMI. However, many of those studies have been focused on testing shielding effectiveness at high electromagnetic frequencies, mostly within the X-band ranges (8.2–12.4 GHz) or even higher frequencies. Commonly used electronic equipment, such as laptops, cell phones, TV sets or radios, emit electromagnetic radiation waves within frequency ranges below 4 GHz. Thus, investigation of EMI shielding performance at low frequencies is also of high importance. In the present work, manganese–zinc ferrite, nickel–zinc ferrite and their combinations were used for fabrication of composite materials with utilization of acrylonitrile-butadiene rubber (NBR). The curing process, physical–mechanical characteristics and EMI absorption shielding efficiency of the composites were investigated at low frequencies. Then, carbon fibres were combined with ferrites with the aim to modify both the tensile characteristics and absorption shielding performance.

Acrylonitrile-butadiene rubber is specialty rubber possessing very good resistance to oil and non-polar solvents. Resistance against chemicals, termo-oxidative stability and mechanical properties increase with increasing amount of acrylonitrile structural units. On the other hand, the increase in acrylonitrile units is connected with a decrease in elasticity. Tailoring of rubber properties can also be achieved by the proper type and content of the filler or fillers applied.

## 2. Experimental

### 2.1. Materials

Manganese–zinc ferrite (MnZn) and nickel–zinc ferrite (NiZn) in powder form were provided by Epcos s.r.o., Czech Republic. Both fillers represent magnetic soft ferrites with spinel structure and similar particle size distribution. Although the total particle size distribution is relatively wide, most particles for both fillers ranged from 10 to 30 μm. The average particle size distribution of both fillers with values D10 and D50 is mentioned in Table 1. Parameters D10 and D50 represent percentage of particles with diameters lower than the given value. D50 can be defined as median, showing that 50% of particles are lower than roughly 16 μm for MnZn ferrite and roughly 21 μm for NiZn ferrite. Carbon fibres (CF) with trademark Carbiso^TM^G were supplied from ELG Carbon Fibre Ltd., Bilston, UK. The average diameter of fibres was 7.5 μm; the length of fibres ranged from 80 to 100 μm. The fibres were surface-covered with silanes (8%). Acrylonitrile-butadiene rubber (NBR) provided by Sibur International, Russia (SKN 3345, content of acrylonitrile 31–35%) served as rubber matrix. Cross-linking of rubber composites was performed by application of sulphur curing system consisting of stearic acid and zinc oxide (Slovlak, Košeca, Slovakia) as activators, N-cyclohexyl-2-benzothiazole sulfenamide (CBS, Duslo, Šaľa, Slovakia) as accelerator and sulphur (Siarkopol, Tarnobrzeg, Poland) as curing agent (Table 2 and Table 3).

### 2.2. Methods

#### 2.2.1. Fabrication and Curing of Rubber Compounds

Two types of rubber composites were fabricated and tested in the study. In the first type of composites, manganese–zinc ferrite, nickel–zinc ferrite and combination of both fillers were incorporated into NBR based matrix. The total content of magnetic fillers was kept on constant level—300 phr. The composite filled only with manganese–zinc ferrite was designated as Mn300; the composite filled only with nickel–zinc ferrite was marked as Ni300. The other three types of composites were designated according to mutual ratio of both fillers, as shown in Table 2. The amount of curing additives was also constant in all composites. The second series of composites were fabricated with the same composition; moreover, carbon fibres were incorporated into rubber compounds in constant loading—25 phr (Table 3). 

Rubber, filler and curing additives were compounded in the chamber of tangential kneading equipment Brabender (Brabender GmbH & Co. KG, Duisburg, Germany) in two step mixing process. The temperature was set to 90 °C and the speed of rotors was 55 rpm. First, rubber was plasticated for 2.5 min, and then stearic acid and zinc oxide were added, and, after next 2 min, magnetic filler or fillers were applied. The total time of first step mixing was 9 min. The rubber compounds were cooled down and calendared in two-roll mill. In the second step, sulphur together with accelerators were introduced and the compounding process continued for 4 min at 90 °C and 55 rpm. Final step was additional homogenization and sheeting of rubber compounds using two-roll mill. The preparation procedure of the second series rubber compounds was very similar, but rubber batch based on NBR and carbon fibres was pre-compounded using semi-industrial kneading machine Buzuluk (Buzuluk Inc., Komárov, Czech Republic). Then, the preparation procedure followed the same conditions as mentioned above. The curing process was performed at 160 °C and pressure of 15 MPa in a hydraulic press Fontijne following the optimum cure time of corresponding rubber compounds. After curing, thin sheets with dimensions 15 × 15 cm and thickness 2 mm were obtained.

#### 2.2.2. Determination of Curing Characteristics

Curing characteristics of rubber compounds were determined from corresponding curing isotherms, which were investigated in oscillatory rheometer MDR 2000 (Alpha Technologies, Akron, OH, USA).

The investigated curing parameters were:

M_L_—minimum torque (dN.m)
M_H_—maximum torque (dN.m)
∆M (dN.m)—torque difference, the difference between M_H_ and M_L_
t_c90_ (min)—optimum curing time that corresponds to torque M_c90_:
  M_c90_ = M_L_ + 0.9∆M (1)
t_s1_ (min)—scorch time
R (dN.m.min^−1^)—curing rate, defined as:
  (2) $$R=\frac{M_{c90}-{\;M}_{s1}}{t_{c90}-{\;t}_{s1}}$$
M_c90_—torque at t_c90_
M_s1_—torque at t_s1_

#### 2.2.3. Investigation of Mechanical Characteristics

The tensile properties of composites were evaluated by using Zwick Roell/Z 2.5 appliance (Zwick Roell Group, Ulm, Germany). The cross-head speed of the measuring device was set to 500 mm.min^−1^ and the tests were carried out in compliance with the valid technical standards. Dumbbell-shaped test specimens (thickness 2 mm, length 80 mm, width 6.4 mm) were used for measurements. Hardness was measured by durometer and expressed in Shore A unit. 

#### 2.2.4. Investigation of Shielding Characteristics

The frequency dependencies of complex (relative) permeability μ = μ′ − jμ″ for toroidal samples were measured using combined impedance/network analysis method by means of a vector analyser (Agilent E5071C) in the frequency range of 1 MHz–6 GHz. During measurements, a toroidal sample was inserted into a magnetic holder (Agilent 16454A) and the complex permeability was evaluated from measured impedances (3):

μ = μ′ − jμ″ = 1 + (*Z* − *Z_air_*)/(j*h*μ_0_
*f* ln(*b*/*c*)) (3)

where *Z* and *Z_air_* are the input complex impedances of the 16454A holder with and without a toroidal sample, respectively, *h* is the height of the sample, μ_0_ = 4π·10^−7^ H/m is the permeability of free space, *f* is the frequency and *b* and *c* are the outer and inner diameters of the sample.

The frequency dependencies of complex (relative) permittivity *ε* = *ε*′ − j*ε*″ for disc samples were measured using combined impedance/network analysis method by means of a vector analyser (Agilent E5071C) in the frequency range of 1 MHz–6 GHz. During measurements, a disc sample was inserted into a dielectric holder (Agilent 16453A) and the complex permittivity was computed from measured admittance (4):

*ε* = *ε*′ − j*ε*″ = (*Y*·*h*)/(jω*ε*_o_S) (4)

where *Y* is the input complex admittance of the 16453A holder with a disc sample, h is the height of the sample, *ε*_0_ = 8.854⋅10^−12^ F/m is the permittivity of free space and S is the area of lower electrode. In case of electrically conductive material with dc electrical conductivity σ_dc_, the imaginary part of *ε* should be replaced by *ε*″ − σ_dc_/2π*ε*_o_. The electrical dc conductivity of composite materials was evaluated using standard two-probe method.

High-frequency single-layer electromagnetic wave absorption properties (return loss RL, matching thickness d_m_, matching frequency f_m_, bandwidth ∆f for RL at −10 dB and RL at −20 dB and the minimum of return loss RL_min_) of composite materials were obtained by calculations of return loss (5):

RL = 20 log |(Z_in_ − 1)/(Z_in_ + 1)| (5)

where Z_in_ = (μ/ε)^1/2^tanh[(jω⋅d/c)(μ⋅ε)] is the normalized value of input complex impedance of the absorber, *d* is the thickness of the single-layer absorber (backed by a metal sheet), c is the velocity of light in vacuum. The composite absorbs maximum of the electromagnetic plane wave energy when normalized value of impedance Z_in_ ≈ 1. The maximum absorption is then reached at a matching frequency f = f_m_, matching thickness *d* = d_m_ and minimum return loss RL_min_. 

## 3. Results and Discussion

### 3.1. Curing Process of Rubber Compounds

**Figure 1:**
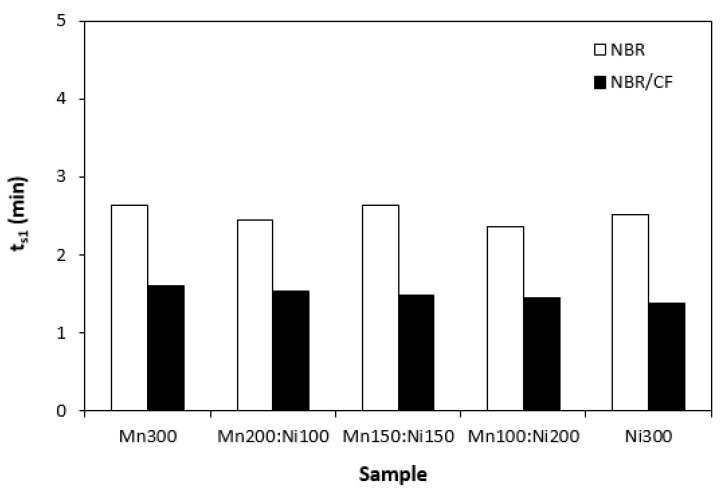
Scorch time t_s1_ of rubber compounds filled with ferrites and composites filled with carbon fibres and ferrites.

**Figure 2:**
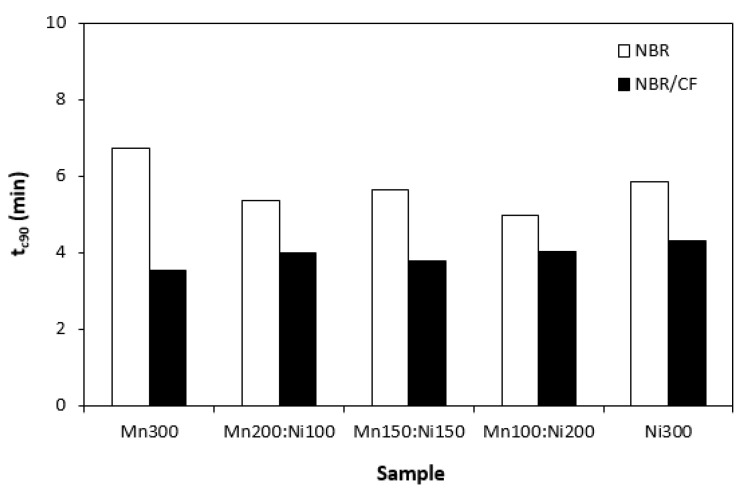
Optimum cure time tc_90_ of rubber compounds filled with ferrites and composites filled with carbon fibres and ferrites.

**Figure 3:**
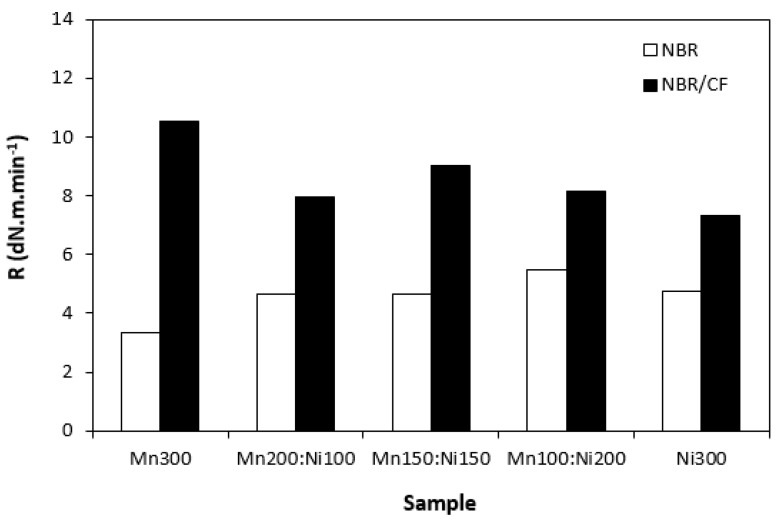
Curing rate R of rubber compounds filled with ferrites and composites filled with carbon fibres and ferrites.

**Figure 4:**
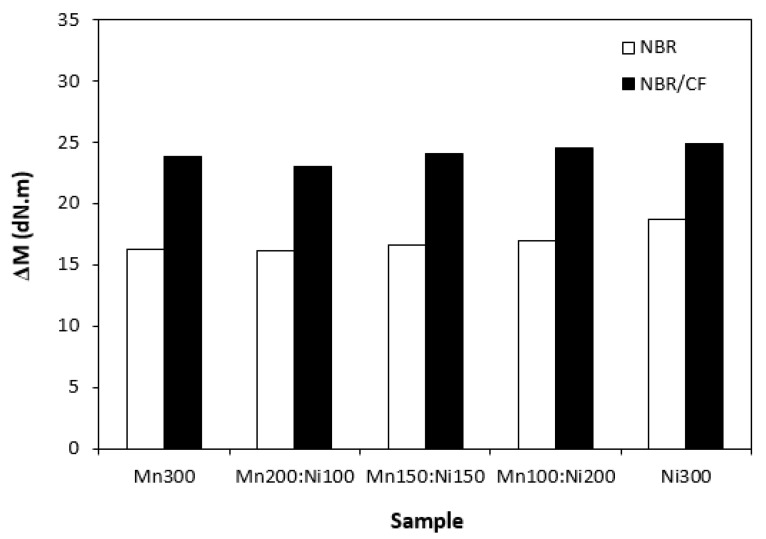
Torque difference ΔM of rubber compounds filled with ferrites and composites filled with carbon fibres and ferrites.

The influence of the tested fillers on curing process of rubber compounds was evaluated based on determination of curing characteristics, scorch time t_s1_ and optimum cure time t_c90._ Then, difference between maximum and minimum torque (ΔM = M_H_ − H_L_) and curing rate R were calculated. As shown in Figure 1, scorch time of rubber compounds filled only with ferrites seems to be independent on type of ferrite or ferrites combinations. Application of carbon fibres caused a decrease in scorch time roughly in 1 min in comparison to the equivalent rubber compounds filled only with ferrites. The influence of ferrites combinations on t_s1_ of composites based on carbon fibres and magnetic fillers seems also to be insignificant. A similar statement can be applied to optimum cure time (Figure 2). Longer optimum cure time was exhibited with rubber compounds filled only with ferrites. The rubber compounds with incorporated carbon fibres required shorter time for their optimum curing, with no clear influence of type of magnetic filler or their combinations. The shorter scorch time and optimum cure time of rubber compounds with added carbon fibres was reflected in a higher curing rate of the corresponding rubber formulations (Figure 3). Based on the achieved results, it becomes apparent that the curing process of CF and ferrites-filled rubber compounds proceeded faster, which can be attributed to the presence of carbon fibres. Carbon-based fillers are characterized by good electrical and thermal conductivity. Thus, presence of CF leads to increased heat transfer within rubber compounds. Composites can thus be heated faster to the curing temperature. Cross-linking reactions proceed faster, which results in overall acceleration of their curing process. From Figure 4, it becomes apparent that rubber compounds based on CF and ferrites exhibited higher differences between maximum and minimum torque ΔM when compared to corresponding rubber compounds filled only with magnetic fillers. This can again be attributed to the presence of carbon fibres, which reinforce and stiffen the rubber matrix. The reinforcing effect of carbon fibres was reflected in the increase in torque increment during the curing process as a result of an increased modulus (see Section 3.3).

### 3.2. Absorption Shielding Performance of Composites

**Figure 5:**
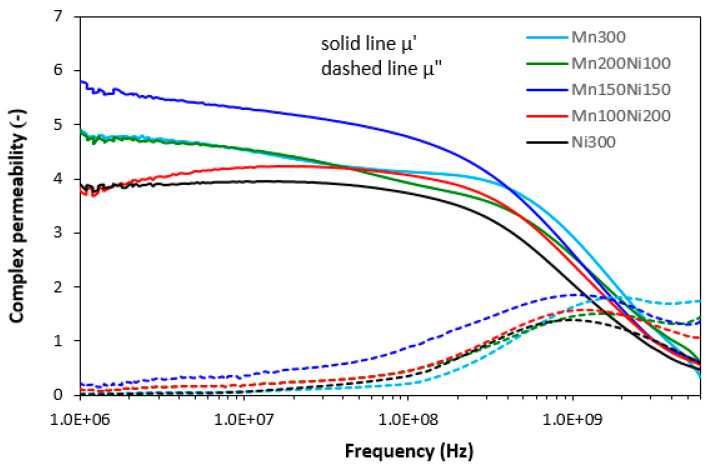
Frequency dependences of complex permeability for composites filled with ferrites.

Commonly used electronic and electromagnetic devices (TV sets, computers, radios, mobile phones, etc.) operate at lower frequencies, usually up to 3–4 GHz. Thus, from a practical point of view, shielding of EMI at low frequencies is of high importance. Absorption shielding efficiency of composites was investigated within the frequency range from 1 MHz to 6 GHz. Complex permeability and complex permittivity were first investigated. Permittivity and permeability have been reported to be crucial parameters influencing EMI shielding efficiency. Complex permeability constitutes the real component and imaginary component (μ = μ′ − jμ″). Real permeability μ′ represents magnetic storage, while imaginary permeability μ″ represents magnetic losses in material. Materials with high permeability have been reported to be good materials for shielding by absorption [18]. Figure 5, depicting frequency dependences of complex permeability for composites filled only with magnetic soft ferrites, demonstrates that the highest real permeability μ′ in all frequency ranges was found for a composite filled with an equivalent ratio of manganese–zinc ferrite and nickel–zinc ferrite (Mn150Ni150). There was recorded almost no change in real part up to roughly 200–300 MHz, and then it sharply decreased with an increase in frequency (from μ′~5.8 in frequency interval 1–100 MHz to μ′ = 0.6 at 6 GHz). The lowest real permeability was exhibited for the composite filled with 300 phr of nickel–zinc ferrite (Ni300). With an increase in frequency, the differences in real permeability of composites filled with ferrites became smaller. Real permeability of all composites decreased to very low values at a maximum frequency. Imaginary permeability μ″ seems to be frequency-independent up to about 100 MHz. Then, it increased to a maximum at a resonance frequency (around 1 GHz) and dropped down. The maximum in μ″(f) dependences corresponds to the maximal permeability loss or magnetic loss. The highest imaginary permeability seems also to be associated with the composite filled with an equivalent ratio of both ferrites (μ″ = 0.24 at an initial frequency, μ″ = 1.86 at a resonance frequency). However, in general, it can be stated that no significant changes in complex permeability were observed depending on type of applied ferrite or ferrites combinations. 

**Figure 6:**
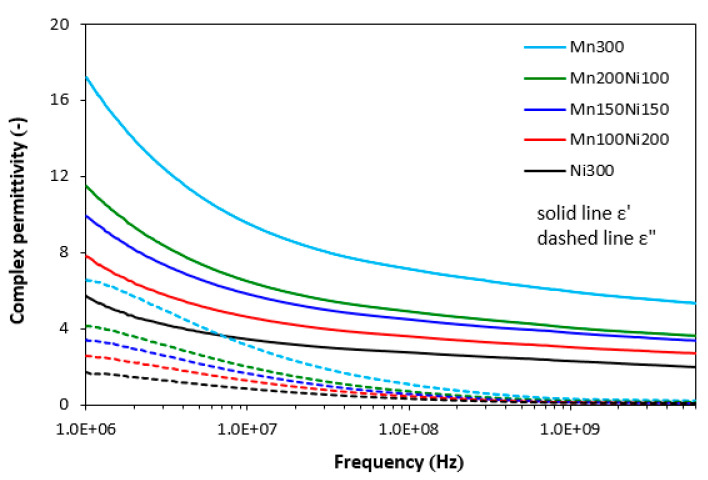
Frequency dependences of complex permittivity for composites filled with ferrites.

Similarly, complex permittivity consists of real and imaginary parts (*ε* = *ε*′ − j*ε*″). Real permittivity *ε*′ or dielectric constant represents electric charge storage capacity, whereas imaginary permittivity *ε*″ indicates dielectric dissipation or losses. As shown in Figure 6, there was clear dependence of complex permittivity on type of ferrite. The highest real permittivity was demonstrated for the composite filled with manganese–zinc ferrite (Mn300), while the lowest was exhibited for the composite filled with nickel–zinc ferrite (Ni300). The largest difference in *ε*′ between both composites was observed at an initial frequency (*ε*′ = 17.2 for Mn300 and *ε*′ = 5.7 for Ni300). As also shown, the higher the proportion of nickel–zinc ferrite, the lower the real permittivity. Real permittivity of composites showed a decreasing trend with an increase in electromagnetic radiation frequency. With increasing frequency, the differences in real permittivity became smaller. At a maximum frequency, the real permittivity of composites Mn300 and Ni300 reached 5.3 and 1.96, respectively. The imaginary permittivity of composites was lower and followed dependences in fillers composition. The composite filled with only manganese–zinc ferrite was found to have the highest *ε*″, while the lowest imaginary part was demonstrated for the composite filled with only nickel–zinc ferrite. The increase in frequency resulted in a decrease in imaginary permittivity up to about 1 GHz and then *ε*″ settled on a constant value with almost no dependence on type of filler or fillers combinations. 

Based on determination of complex permeability and complex permittivity, return loss RL in decibels was calculated. Return loss refers to the amount of electromagnetic radiation plane wave that can be efficiently absorbed by a shielding material. It has been reported in scientific studies that materials showing return loss at −10 dB can efficiently absorb roughly 90–95% of EMI. Almost 99% of EMI can be absorbed by a shield reaching return loss at −20 dB [40,41,42]. 

**Figure 7:**
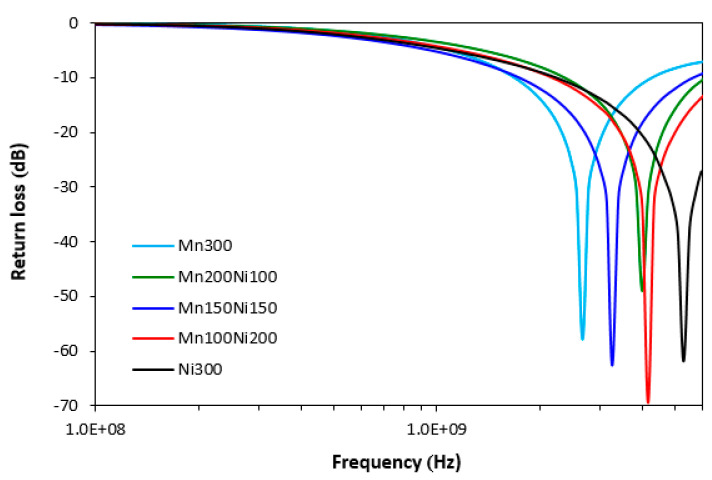
Frequency dependences of return loss for composites filled with ferrites.

The frequency dependences of return loss for magnetic soft ferrites filled composites are graphically illustrated in Figure 7. The calculated values of electromagnetic absorption shielding characteristics, i.e., minimum value of return loss RL_min_ (maximum absorption shielding performance) at a matching frequency f_m_, matching frequency f_m_ and effective absorption frequency bandwidth Δf for RL at −10 dB and −20 dB, are summarized in Table 4. As shown in Figure 7 and Table 4, with increasing content of nickel–zinc ferrite in fillers combinations, absorption maxima and absorption shielding effectiveness of composites shift to higher frequencies of EMI. The absorption maximum of the composite filled with manganese–zinc ferrite (Mn300) was −58 dB at matching frequency 2660 MHz, while the composite filled with nickel–zinc ferrite (Ni300) achieved an absorption maximum of −62 dB at matching frequency 5260 MHz. The composite loaded with 300 phr of MnZn ferrite exhibited the lowest effective frequency bandwidth at −10 and −20 dB (RL at −10 dB within 1700–4200 MHz frequency range and RL at −20 dB within 2320–3060 MHz frequency range), which means that this composite is the least effective for EMI absorption shielding. On the other hand, the best absorption shielding material can be considered the composite filled with 300 phr of nickel–zinc ferrite (Ni300) as it demonstrated return loss at −10 dB and −20 dB within the widest frequency ranges, i.e., from 2250 MHz to 6 GHz at −10 dB and from 3910 MHz to 6 GHz at −20 dB. It must be noted that, due to limitations of the used equipment and possibility to measure only up to 6 GHz, the effective frequency absorption bandwidth for this composite would be much wider. From Figure 7 and Table 4, it becomes obvious that effective absorption frequency ranges at RL = −10 dB and −20 dB become broader with increasing ratio of nickel–zinc ferrite, which suggests that the NiZn filler demonstrates better absorption shielding potential. However, based on the achieved results, all the composites show satisfactory absorption shielding potential as they reached return loss at −10 dB and −20 dB within broad frequency ranges. 

**Figure 8:**
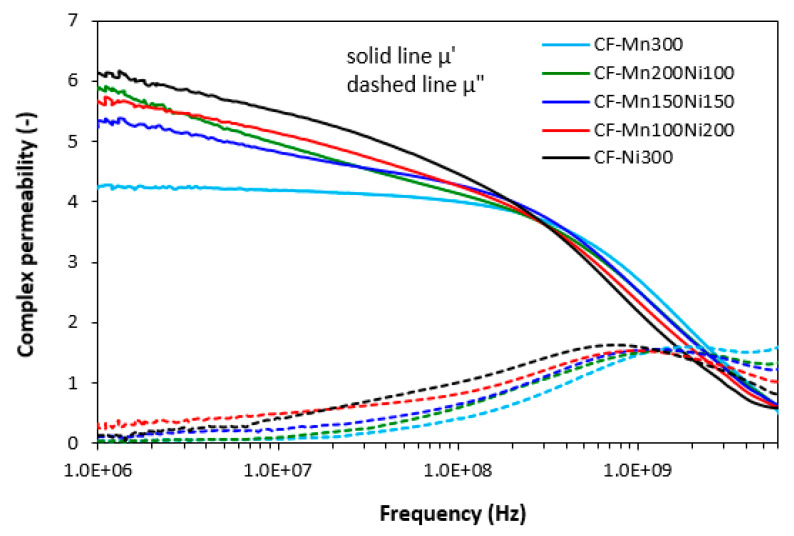
Frequency dependences of complex permeability for composites filled with carbon fibres and ferrites.

**Figure 9:**
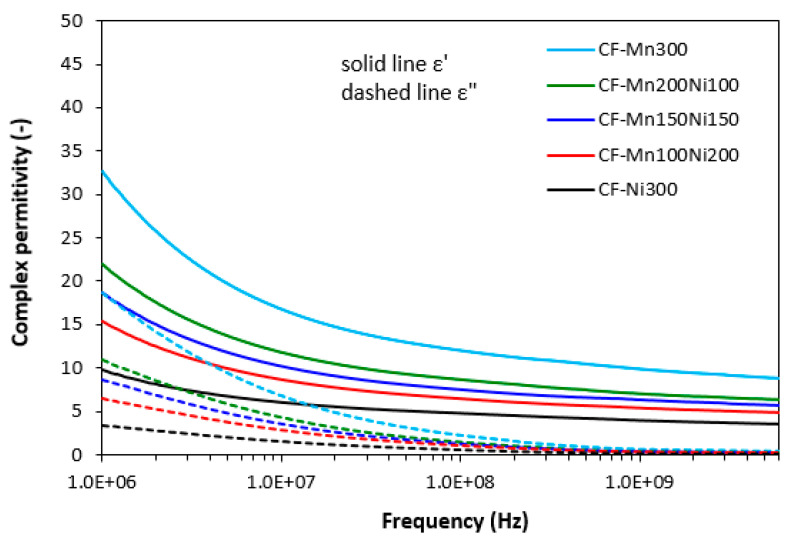
Frequency dependences of complex permittivity for composites filled with carbon fibres and ferrites.

**Figure 10:**
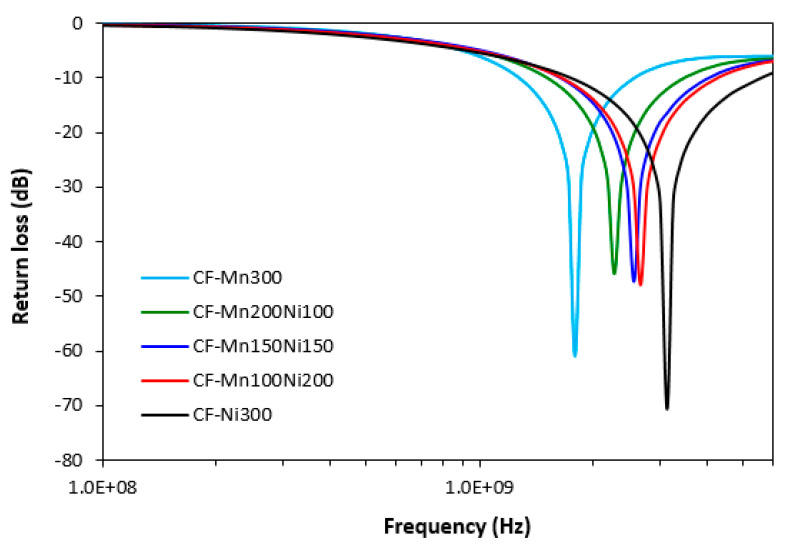
Frequency dependences of return loss for composites filled with carbon fibres and ferrites.

The graphical dependences of complex permeability, complex permittivity and return loss for composites filled with carbon fibres and magnetic soft ferrites are presented in Figure 8, Figure 9 and Figure 10, and the computed values of electromagnetic absorption shielding characteristics, minimum value of return loss RL_min_ at a matching frequency f_m_, matching frequency f_m_ and effective absorption frequency bandwidth Δf for RL at −10 dB and −20 dB are summarized in Table 5. From Figure 8, the highest real permeability at the initial frequency was exhibited for the composite filled with CF and nickel–zinc ferrite (CF-Ni300), while the lowest real permeability was exhibited for the composite based on CF and manganese–zinc ferrite (CF-Mn300). The real permeability of CF and ferrites filled composites showed slight decreasing trends up to 300 MHz and then declined to very low values at maximum frequency. Simultaneously, the differences in real permeability among composites became less visible. There were recorded almost no differences in imaginary permeability of composites in dependence on ferrite or ferrites combinations. Imaginary permeability of composites was lower than the real part, reached a maximum at a resonance frequency (around 1 GHz) and then declined. When comparing Figure 5 and Figure 8, one can see that the frequency dependences of complex permeability for composites filled with carbon fibres and ferrites were comparable with those for the composites filled only with ferrites.

From the frequency dependences of complex permittivity for hybrid composites (Figure 9), the highest real part *ε*′ in all frequency ranges was demonstrated by the composite filled with CF and manganese–zinc ferrite. The recorded value of *ε*′ for this composite was 32.7 at 1 MHz. Then, it decreased to 8.8 at 6 GHz. Further, the higher the proportion of nickel–zinc ferrite, the lower the real part. With a rise in frequency, differences in real permittivity became smaller. The composite filled with CF and nickel–zinc ferrite was found to have the lowest *ε*′, which also seemed to be the least dependent on the frequency. By increasing frequency from 1 MHz to 6 GHz, the real part decreased from 9.9 to 3.5. Similar dependences on type of ferrite or ferrites combinations were recorded for imaginary permittivity, which was lower than the real part. The differences in imaginary permittivity were negligible over 1 GHz. Comparing Figure 6 and Figure 9, it is apparent that the real permittivities of composites filled with carbon fibres and magnetic fillers were about two times higher when compared to equivalent composites filled only with ferrites. Similarly, the imaginary permittivities of composites with incorporated carbon fibres were higher than those of composites filled only with ferrites at low frequencies. With an increase in frequency, the differences in *ε*″ for both composite types became less visible.

Frequency dependences of return loss for composites filled with carbon fibres and ferrites (Figure 10) demonstrated very close dependence on the type of magnetic filler or ferrites combinations, but they were influenced by presence of CF. It can be stated that the most efficient EMI absorption shield can be considered the composite filled with CF and nickel–zinc ferrite (CF-Ni300). As shown in Figure 10 and Table 5, this composite absorbs electromagnetic radiation within the widest frequency ranges (from 1700 to 5500 MHz at RL = −10 dB and from 2620 MHz to 3700 MHz at RL = −20 dB). It also exhibited the lowest peak, representing the maximum absorption shielding performance (RL_min_ = −71 dB), reaching matching frequency f_m_ = 3130 MHz. On the other hand, the composite with designation CF-Mn300 demonstrated the narrowest frequency bandwidth for absorption shielding at −10 and −20 dB. The effective absorption frequency bandwidth moved from 1260 MHz to 2560 MHz at −10 dB (Δf = 1300 MHz) and from 1600 MHz to 1970 MHz at −20 dB (Δf = 370 MHz). The absorption maximum of this composite was −61 dB at a matching frequency 1783 MHz. From Table 5, the higher the proportion of nickel–zinc ferrite in ferrites combinations, the broader the effective absorption frequency bandwidths for composite materials. Simultaneously, the increasing amount of nickel–zinc ferrite resulted in shifting of composites absorption shielding performance to higher frequencies of EMI. When comparing Figure 7 and Figure 10 and Table 4 and Table 5 composites filled with CF and ferrites demonstrated lower matching frequencies, narrower effective absorption frequency bandwidths Δf for RL at −10 dB and −20 dB and narrower absorption peaks. This means that combination of carbon fibres with magnetic fillers resulted in shifting of absorption shielding ability of composites to lower frequencies of EMI on hand. On the other hand, it becomes apparent that absorption shielding efficiency of composites based on CF and ferrites was lower based on their narrower absorption peaks.

**Figure 11:**
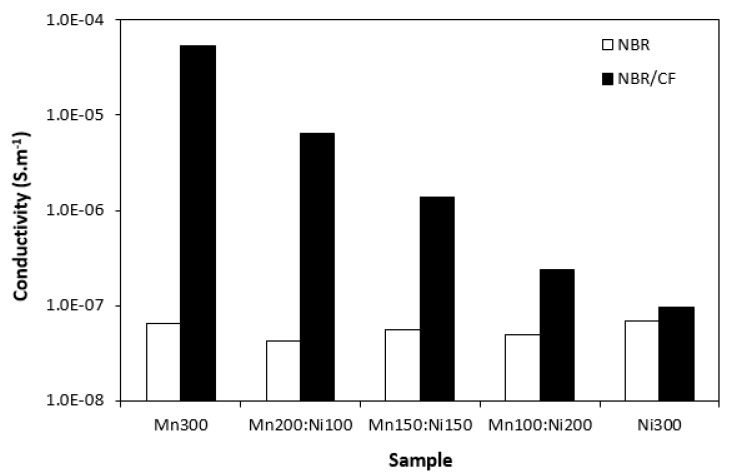
Conductivity of composites filled with ferrites and composites filled with carbon fibres and ferrites.

To better understand the influence of the tested fillers on absorption shielding efficiency, the electrical conductivity of composites was investigated. From Figure 11, it can be observed that electrical conductivity of composites filled only with ferrites was low and seemed not to be influenced by type of ferrite or ferrites combinations. Incorporation of carbon fibres resulted in an increase in electrical conductivity. Carbon-based fillers, including carbon fibres, are characterized by unique electrical conductivity, and thus the presence of CF in composites caused an increase in electrical conductivity. From Figure 11, electrical conductivity of hybrid composites showed an increasing trend with increasing content of manganese–zinc ferrite in magnetic fillers combinations. The differences in electrical conductivity between both composite types were less visible with increasing proportion of nickel–zinc ferrite. Higher electrical conductivity of composites filled with carbon fibres and ferrites was reflected in their higher complex permittivity. As already mentioned, real permittivity represents electric charge storage in the material and is related mainly to amount of micro-capacitors and polarization mechanisms [43]. Polarization of the conducting filler, rubber matrix as well as filler–rubber interfacial polarization happens depending on frequency range [44,45]. Conductive carbon fibres act as micro-capacitors and contribute to formation of localized charges within composite systems. Due to the cylindrical structure of CF with high aspect ratio, the distance between the particles in the rubber matrix is reduced, which results in higher filler–rubber interfacial charge polarization. Imaginary permittivity correlates with dissipation of electrical energy (dielectric dissipation or losses). It is attributed to polarization mechanisms: interfacial polarization, electronic and dipole polarization, natural resonance and relaxation phenomena [46,47]. As suggested, presence of CF and high amount of interfaces in the rubber matrix increases interfacial filler–rubber polarization and leads to generation of dipoles on semi-conductive ferrite particles. This results in higher imaginary permittivity and, subsequently, higher complex permittivity. Among all the tested materials, the highest real and imaginary permittivity was found regarding the composite filled with CF and manganese–zinc ferrite (CF-Mn300) with the highest electrical conductivity. The increase in electrical conductivity for hybrid composites with increasing amount of manganese–zinc ferrite points to higher electrical conductivity of manganese–zinc ferrite. However, this was not experimentally confirmed by the investigation of electrical conductivity for composites filled only with ferrites. An enhanced synergic effect in conductivity when combining carbon fibres and manganese–zinc ferrite should be considered. 

Following the achieved results, in can be summarized that electrical conductivity is a very important parameter that influences absorption shielding performance of composites, mainly at low frequencies. The higher the electrical conductivity of composites, the lower EMI they absorbed. It has been described in literature sources that materials with high electrical conductivity are susceptible to shield EMI by reflection, mainly at low frequencies [48,49,50]. The experimentally obtained results correlate very well with those assumptions. It can be stated that composite filled with 300 phr of nickel–zinc ferrite (Ni300) is the best absorption composite shield as it demonstrated the widest frequency absorption bandwidth at RL = −10 and −20 dB and the widest absorption peak.

### 3.3. Physical–Mechanical Properties of Composites

**Figure 12:**
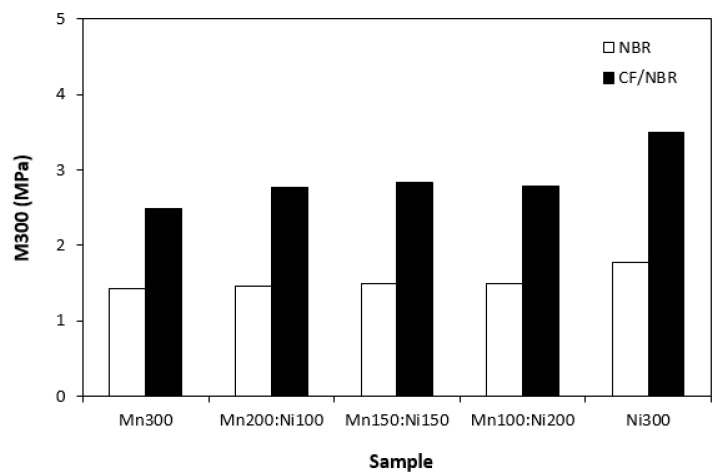
Modulus M300 of composites filled with ferrites and composites filled with carbon fibres and ferrites.

**Figure 13:**
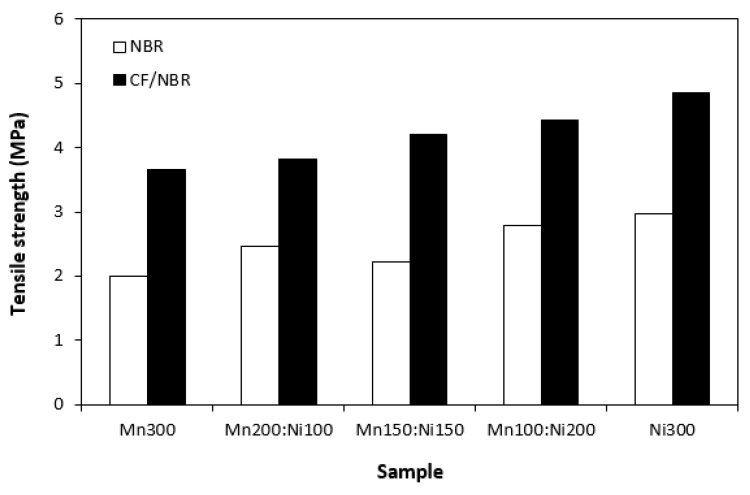
Tensile strength of composites filled with ferrites and composites filled with carbon fibres and ferrites.

**Figure 14:**
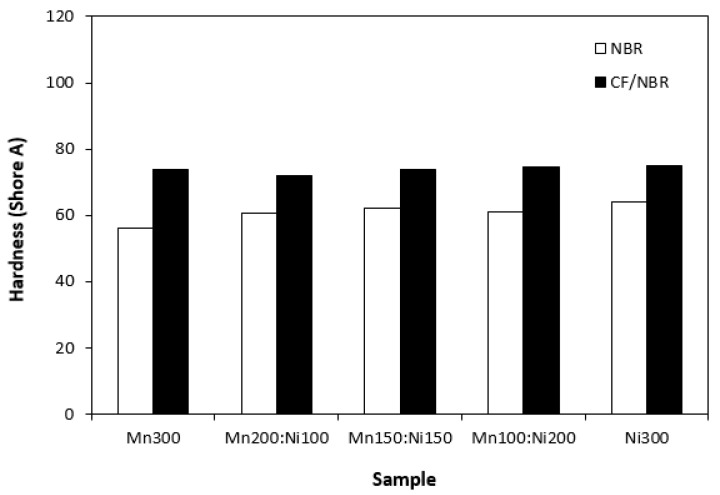
Hardness of composites filled with ferrites and composites filled with carbon fibres and ferrites.

**Figure 15:**
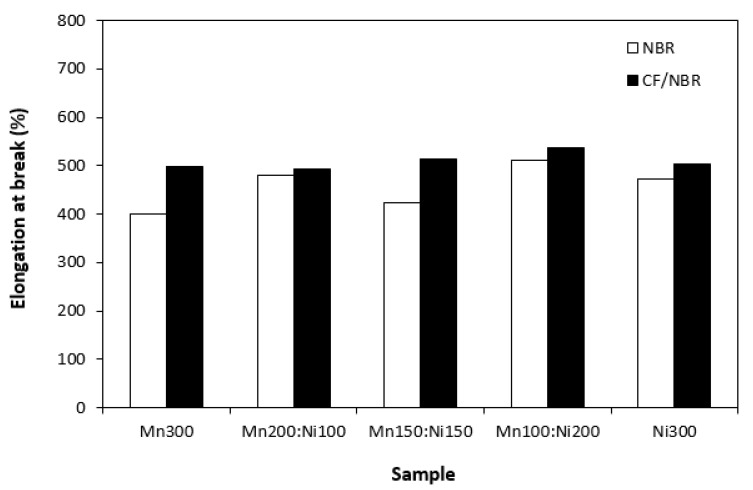
Elongation at break of composites filled with ferrites and composites filled with carbon fibres and ferrites.

The physical–mechanical characteristics of composites are graphically illustrated in Figure 12, Figure 13, Figure 14 and Figure 15. It becomes apparent that modulus M300 (Figure 12) and tensile strength (Figure 13) of composites filled only with ferrites were low. As ferrites are stiff metal-based powdery materials with relatively wide particle size distribution, they do not reinforce the rubber matrix; thus, low tensile characteristics were expected. Incorporation of carbon fibres resulted in an increase in both modulus and tensile strength. Carbon-based fillers are also characterized by unique structure that enables good physical or physical-chemical adhesion with rubber matrices, which results in improved tensile behaviour of composite materials. When looking at Figure 13, tensile strength of both composite types showed a slight increasing trend with increasing content of nickel–zinc ferrite in ferrites combinations. The composite filled with 300 phr of manganese–zinc ferrite (Mn300) was found to have a tensile strength of 2 MPa, while the composite filled only with nickel–zinc ferrite (Ni300) exhibited a tensile strength of 3 MPa. By incorporation of carbon fibres, the tensile strength increased from about 3.5 MPa for the composite with designation CF-Mn300 to almost 5 for composite CF-Ni300. This suggests that nickel–zinc ferrite shows a slightly higher reinforcing effect when compared with manganese–zinc ferrite. Nickel–zinc ferrite has narrower particle size distribution. The lower the particle size, the higher the specific surface area and the higher the reinforcing effect. 

Hardness of composites seems not to be dependent on type of ferrite or ferrites combinations (Figure 14). Higher hardness of composites with incorporated CF is a logical reflection of the higher overall content of fillers in the rubber matrix and higher stiffening effect of carbon fibres. Composites based on CF and ferrites exhibited slightly higher elongation at break when compared to equivalent composites filled only with magnetic fillers. As also shown in Figure 15, elongation at break of both composite types fluctuated in the low range of experimental values independent of type of ferrite or ferrites combinations.

## 4. Conclusions

In this study, composites based on magnetic soft ferrites and composites based on carbon fibres and ferrites were fabricated and tested for EMI absorption shielding. 

The results revealed that scorch time and optimum cure of both composite types were not influenced by type of ferrite or ferrites combinations. Incorporation of carbon fibres caused increased heat transfer within rubber compounds. The faster heating up to curing temperature resulted in overall acceleration of the curing process of composites based on carbon fibres and magnetic fillers. Both composite types showed absorption peaks, which suggests that they can absorb harmful electromagnetic radiation within specific frequency ranges. With increasing content of nickel–zinc ferrite in magnetic fillers combinations, absorption maxima and absorption shielding ability shift to higher frequencies of EMI. Simultaneously, effective absorption frequency bandwidths become broader, which suggests that nickel–zinc ferrite demonstrates better absorption shielding potential. Composites filled with carbon fibres and ferrites exhibited absorption shielding performance at lower frequencies. Their absorption shielding ability was lower when compared to composites filled only with ferrites as a result of their higher electrical conductivity and, thus, higher permittivity. On the other hand, carbon fibres reinforced the rubber matrix, which caused improvement in physical–mechanical properties of hybrid composites. The lower particle size distribution of nickel–zinc ferrite led to a slight increase in tensile strength for composites with increasing nickel–zinc ferrite ratio.

## Figures and Tables

**Table 1:** Structural characteristics of ferrites.

Filler	Particle Size Distribution	D10	D50
MnZn	0.7–50 μm	4.7 μm	16.3 μm
NiZn	0.2–70 μm	3.0 μm	21.4 μm

**Table 2:** Composition of composites filled with ferrites in phr and their designation.

NBR	100	100	100	100	100
ZnO	3	3	3	3	3
stearic acid	2	2	2	2	2
CBS	1.5	1.5	1.5	1.5	1.5
sulphur	1.5	1.5	1.5	1.5	1.5
MnZn ferrite	300	200	150	100	0
NiZn ferrite	0	100	150	200	300
designation	Mn300	Mn200 Ni100	Mn150 Ni150	Mn100 Ni200	Ni300

**Table 3:** Composition of composites filled with carbon fibres and ferrites in phr and their designation.

NBR	100	100	100	100	100
ZnO	3	3	3	3	3
stearic acid	2	2	2	2	2
CBS	1.5	1.5	1.5	1.5	1.5
sulphur	1.5	1.5	1.5	1.5	1.5
Carbon fibres	25	25	25	25	25
MnZn ferrite	300	200	150	100	0
NiZn ferrite	0	100	150	200	300
designation	CF-Mn300	CF-Mn200 Ni100	CF-Mn150 Ni150	CF-Mn100 Ni200	CF-Ni300

**Table 4:** Electromagnetic absorption parameters of composites filled with ferrites.

Sample	RL_min_ (dB)	f_m_ (MHz)	Δf (MHz) −10 dB	Δf (MHz) −20 dB
Mn300	−58	2660	2500	740
Mn200Ni100	−49	3980	3600	1160
Mn150Ni150	−63	3250	3800	1130
Mn100Ni200	−69	4140	3800	1530
Ni300	−62	5260	3750	2090

**Table 5:** Electromagnetic absorption parameters of composites filled with carbon fibres and ferrites.

Sample	RL_min_ (dB)	f_m_ (MHz)	Δf (MHz) − 10 dB	Δf (MHz) − 20 dB
CF-Mn300	−61	1783	1300	370
CF-Mn200Ni100	−46	2267	1950	540
CF-Mn150Ni150	−47	2556	2380	660
CF-Mn100Ni200	−48	2661	2650	740
CF-Ni300	−71	3130	3800	1080

## Data Availability

Data Availability Statements are available in section “MDPI Research Data Policies” at https://www.mdpi.com/ethics.
